# Evaluation of Participant Success in Gamified Drone Training Simulator Using Brain Signals and Key Logs

**DOI:** 10.3390/brainsci11081024

**Published:** 2021-07-31

**Authors:** Durmuş Koç, Ahmet Çağdaş Seçkin, Zümrüt Ecevit Satı

**Affiliations:** 1 Informatics Department, Institute of Graduate Studies in Sciences, Istanbul University, Istanbul 34134, Turkey; 2 Computer Programming Department, Vocational School of Karahallı, Uşak University, Uşak 64000, Turkey; 3 Department of Computer Engineering, Adnan Menderes University, Aydın 09010, Turkey; seckin.ac@gmail.com; 4 Department of Business Administration, Istanbul University, Istanbul 34452, Turkey; zsati@istanbul.edu.tr

**Keywords:** aerospace simulation, human computer interaction, data processing, educational technology

## Abstract

The risk of accidents while operating a drone is quite high. The most important solution is training for drone pilots. Drone pilot training can be done in both physical and virtual environments, but the probability of an accident is higher for pilot trainees, so the first method is to train in a virtual environment. The purpose of this study is to develop a new system to collect data on students’ educational development performance of students during the use of Gamified Drone Training Simulator and objectively analyze students’ development. A multimodal recording system that can collect simulator, keystroke, and brain activity data has been developed to analyze the cognitive and physical activities of participants trained in the gamified drone simulator. It was found that as the number of trials increased, participants became accustomed to the cognitive load of visual/auditory tasks and therefore the power in the alpha and beta bands decreased. It was observed that participants’ meditation and attention scores increased with the number of repetitions of the educational game. It can be concluded that the number of repetitions lowers stress and anxiety levels, increases attention, and thus enhances game performance.

## 1. Introduction

The first use of Unmanned Aerial Vehicles (UAVs) was defense systems, but nowadays it has become widespread for civilian purposes. Today, drones are used for entertainment and media [1], search and rescue missions [2], surveillance [3], mapping [4], agriculture [5], traffic monitoring [6] and disaster management [7]. Today, UAVs used for civilian purposes are often called as drones. When talking about drones, ordinary people first think of small-scale quadcopters. There are several definitions for drones, but Clarke, who defines devices that are heavier than air, capable of continuous and reliable flight, do not carry people and can be controlled enough to perform useful functions, tried to provide a more comprehensive definition [8]. Drone piloting is expected to be a popular profession in the near future, according to the 2018 report of the World Economic Forum [9]. As in all areas where there is a human factor, there are risks of material and moral accidents during remote drone control [8,10]. To minimize these risks, various institutions and organizations offer many regulations and drone-pilot training. In fact, many countries require pilots to have pilot certification for use of drones and subject their journey to various legal restrictions [11]. Drone training can be done in both physical and virtual environments, but pilot candidates are more likely to have an accident, so the first method is training in a virtual environment [12].

Computer-based simulation programs are preferred for operator and pilot training because they can minimize costly, complex, and risky real-life situations. Scenario-based training environments allow processes to be easily changed, improved, and repeated. Many studies show that working in such environments has a positive effect on student learning performance [13,14]. It is also possible to change the training scenarios used in education to increase motivation in desired tasks and activities. The main reasons behind this high motivation and increased pleasure are human emotions felt during games such as scoring, gaining status, competition, puzzle solving, and social proof [15,16]. Points or ranking lists are used to increase player motivation, improve competition between players and groups of players, and make games more enjoyable [16].

It is called flow theory to adjust the most appropriate cognitive load by providing an engaging experience at an optimum level in teaching environment and game designs [17]. Regarding the changes caused by video games in the brain, researchers mostly focused on neuro-cognitive issues related to attention, visual-spatial skills, cognitive workload, cognitive control, skill acquisition, and reward processing [18]. In an experimental study on the relationship between game and EEG, it was concluded that increasing game experience can provide faster learning and game designs for player performance and can be performed with neuro measurements [19].

When the studies on the performance evaluation of simulation environments are examined, it is seen that research methods such as meta-analysis [20], questionnaire [21,22], content analysis [23], observation and interview [24] are used more. Meta-analysis is the re-analysis of the results by estimating the effect size in order to obtain a general result by examining the current study results related to the research subject in the literature [25,26]. The survey method is carried out by collecting the answers to the questions determined about the research from the participants. This method has some difficulties, such as the fact that the answers of the participants may vary according to their current mental state and the participants cannot enter the research in depth. This method, which is often criticized, is widely used to determine the characteristics and relationships of sociological and psychological variables [27]. Content analysis method is to summarize, compare and interpret all data in sources such as communication materials, written materials, images, visual and audio recordings by making a diagram [28]. The observation method is the collection and analysis of comprehensive and detailed research data from the experience of the participants in a specific field, without time limitation [29]. The interview method is carried out by asking questions to the participants to collect the data required for the research and taking note of the collected data and then analyzing these data [30].

In addition to traditional methods of observing changes in the educational environment, more objective methods have gained popularity in recent years. The most popular is the observation and understanding of variables in an educational environment with a variety of sensors. In a study conducted in 2015, it was observed that the brain activity of a group of students was collected simultaneously for the first time using EEG titles and the effect of various teaching methods on attention was measured [31]. In another study they conducted as a continuation of this study, the researchers found a relationship between student participation and attention [32]. In a study examining the effect of colors on the heart rhythm of students in the classroom, it was found that light colors have a more positive effect [33]. In a different study using MRI and fMRI, the positive effect of game-based learning on the learning process was demonstrated [34]. In another study, it was revealed that video games increase player processing speed, hand-eye coordination, reduce reaction time and player stress level [35]. In a study on the detection of emotion intensities in games, it is seen that it is estimated by heart rate (HR) and facial expressions (FE) [36]. In a study on virtual reality games, the emotional states of individuals playing the game were examined using Electroencephalography (EEG), Galvanic skin response (GSR), and heart rate [37]. Information about these studies is presented in Table 1.

When studies examining the relationship between EEG and simulation environments were examined, it was stated that the alpha band power gradually decreased in the explorations after the first visual exploration [38]. Repetitive behaviors have been reported to reduce stress and anxiety [39,40]. It is stated that decreasing stress and anxiety increases attention [41].

In this study, it is aimed to investigate whether objective methods are possible to identify successful individuals during education and to create an experimental setup for this purpose. In the study, it was aimed to provide simulator training as an objective measurement method and to collect time, number of keystrokes and EEG signal data simultaneously during training. Unlike existing empirical studies, this study is expected to provide the following contributions to the literature with multimodal and objective applications.

- Is it possible to identify successful people in the educational environment by simultaneously recording and analyzing the number of keystrokes or brain signals?
- What effect does the number of training trials have on human performance?
- Is there a certain relationship in the performance and mental activities of the participants during the training repetitions in the simulator?
- Is there a relationship between performance in training trials and attention and meditation metrics?

## 2. Materials and Methods

**Figure 1:**
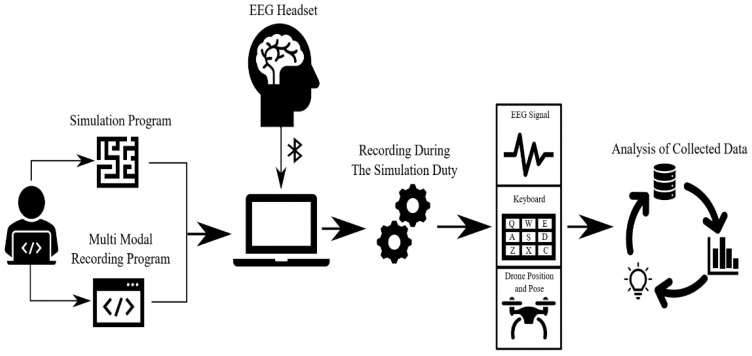
General method flowchart.

The method used for the purpose of this article is depicted in Figure 1. The first task in the study is the creation of Gamified Drone Training Simulation (GDTS). A recording system was created that collects simulator, keyboard, and brain activity data to examine the effect of brain signals and keyboard logs on user training performance during GDTS use. Then GDTS and recording software was installed on the training computer. In addition to the standard equipment of the computer where the training will be made, a mobile electroencephalography (EEG) headset was connected to the computer to record brain signals via Bluetooth. All data were recorded simultaneously thanks to the multi-threading software prepared. In the last stage, the data were analyzed.

### 2.1. Drone Simulator

**Figure 2:**
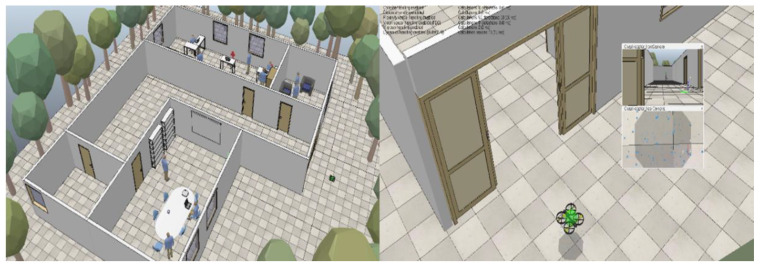
Simulation environment.

CopelliaSim (V-Rep) simulator was used for gamification in the study. The track shown in Figure 2 is designed on the simulator. The “quadricopter” mobile robot model in the V-Rep simulator was used for drone simulation. The Python compatible version of the Remote-API of the simulator program was used to translate control commands received from the computer keyboard into clear commands with the simulator setting. The simulation can be started and stopped with the Remote API, and all kinds of information in the simulation can be collected at certain intervals. Elements in the simulator system work in Lua language. There is an internal control command code written in Lua language for drone flight control. This flight control code only allows the drone to control the propeller rotation speeds and obey the given steering commands. With the commands received on the keyboard, the quadcopter model is moved to the target point in the simulator and the drone movement control is performed. User commands received from the keyboard are presented in Table 2. The drone model has two cameras facing forward and downward. Separate command keys are assigned to take photos from these cameras, as shown in Table 2.

Except for the drone model, doors and people can move within the track. When the simulation starts, the doors open automatically. Three human models move at random speed and direction within the building. Human models avoid all objects in their immediate surroundings during their movements.

The task that the participants are asked to complete in the course is to take pictures of the computers in the building with the sub-camera and take pictures of the occupants with the front-facing camera. Participants saw the GDTS chambers and target’s location prior to the mission. During the game, the participants were tasked to take photos of the targets without hitting or dropping the drone. When the studies on games and education were examined, it was seen that the number of game playing and training repetitions was generally determined as 3 [42,43,44]. Therefore, each participant was asked to repeat the same task 3 times during the experiments.

**Figure 3:**
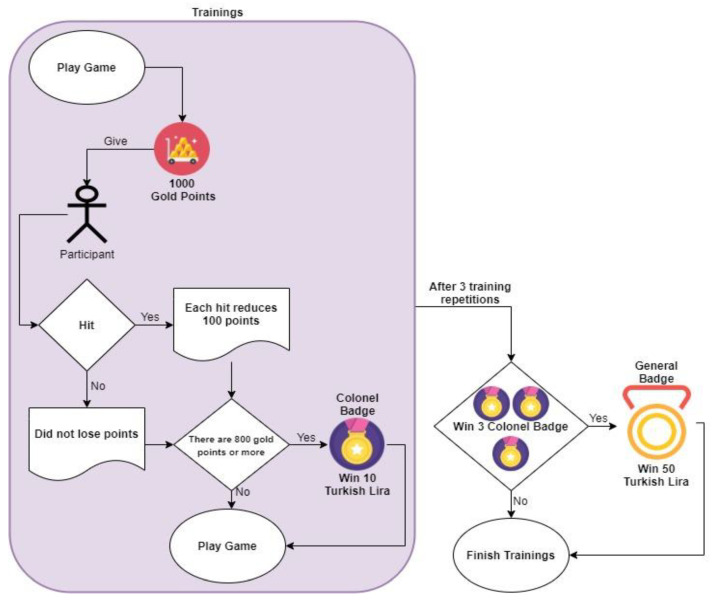
Game flow and game score relationship flowchart.

The simulator environment has been gamified in the study. The flow chart of the game and the game score relationship flow chart is presented in Figure 3. At the beginning of the game, participants were given a symbolic 1000 gold points. Each hit in the game will result in a loss of 100 gold points. Each game replay starts with the current 1000 gold points without any changes. Participants who completed the task with more than 800 gold points after each track repeat were awarded 1 Colonel Badge. Each colonel badge is equivalent to 10 Turkish Lira (TL) shopping vouchers taken from the cafe where the laboratory is located. When all replays were completed, participants who earned a total of 3 Colonel Badges received 1 General Badge in exchange for these badges. The General badge is equivalent to the right to shop at the cafe for 50 TL.

### 2.2. Recording Software

**Figure 4:**
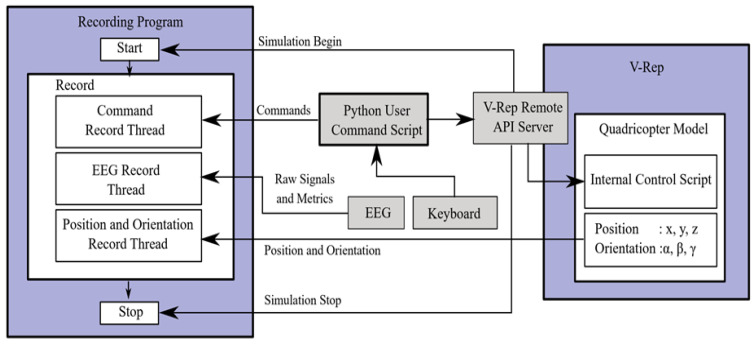
Recording program and simulation environment interaction.

The flowchart showing the data collection processes and interaction with the simulation environment is depicted in Figure 4. In the data collection program, the initial triggering of the keyboard, EEG and simulator listener tasks is performed with the main program. In the recording program, all listener tasks are run as separate threads. The recording program listens to the simulation program at the beginning. With the start of the simulation, the main program starts all listener threads and ends when the simulation is finished. A timestamp is used on each recorded data. The keyboard listener records all commands applicable to the drone. The simulator listening threads record the drone’s 3-axis position (*x*, *y*, *z*) and pose angles (α, β, γ) 10 times per second in the simulator. Neurosky Mindwave Mobile headset was used as the EEG header. Raw signal, attention and meditation metrics are taken from EEG headsets. The raw signal is sampled at 512 Hz, and attention and meditation data are acquired at 1 Hz. Attention and meditation emotion values are measurements produced by the manufacturer from the raw signal, these measurements range from 0 to 100 [45,46].

### 2.3. Analysis of Collected Data

The data collected during the use of GDTS in the study were analyzed separately for each participant according to the order of the task. The analysis provides a timed breakdown of the number of times a participant hit the control keys from the first to the last task, changes in EEG signals and emotional measurements, and changes during training sessions. Time-frequency analysis is adopted for the analysis of the raw EEG signals. For this reason, the Short Time Fourier Transform (STFT) method was applied to a single channel EEG signal of 1 s length, and the spectral power change amounts in the frequency bands presented in Table 3 were analyzed for EEG signals [39,47].

## 3. Experiments and Discussion

Participants of the study were randomly selected on a voluntary basis among individuals aged 18–32. Before the experiments, the participants were informed about the registration system and the experimental procedure, and informed consent was obtained from the participants. The experiments were carried out in an environment free of noise as much as possible, at room temperature, and the participants could keep their mobile phones turned off during the experiments. In the experiments, the participants are expected to repeat the task defined in GDTS three times. The task involves participants taking photos of the computers in the building via the sub-camera and the occupants via the front camera. During the training, participants were asked to take photos of the targets without causing the drone to fall or stop. The trials were completed with each participant repeating the same task 3 times. The information about the age, education level and game experience of the participants was obtained. This information is presented in Table 4.

**Figure 5:**
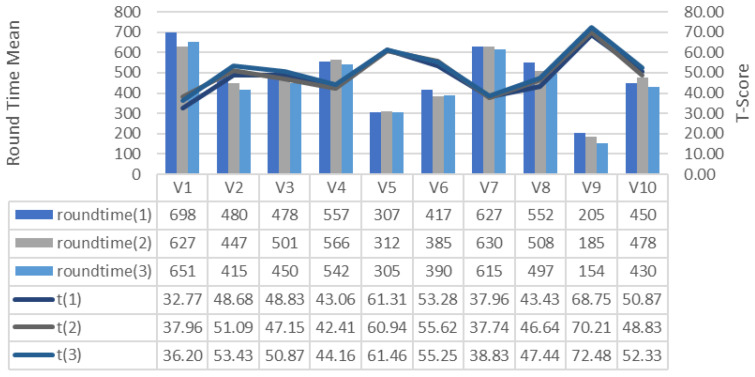
Relationship between GDES average track completion time and game performance (T-Score).

Game experience indicates whether the participants played games for at least 1 h a week in the last 1 month. T-scores are presented in Figure 5 according to how long the participants completed the course and the duration of the tour completion between participants. The time users spend to complete the course usually decreases after each repetition. It is the V9 that completes the course the fastest. Realizing the shortest completion time, the V9 achieved the record of 154 s on the third trail. Task Completion Times of the participants were used for analysis. The Z-score given in Equation (1) was calculated initially to complete the course as quickly as possible. This means higher performance. The x value is the time for a participant to complete that round, µ is the average time for all participants to complete that round, and σ is the standard deviation of the round completion time for all participants. The T-score given in Equation (2) was calculated using the Z-score. In the research findings, the T-Score value of each training repetition was expressed as t (1), t (2) and t (3).

Z-score = (x − µ)/σ(1)

T-score = 50 + 10 × Z-score(2)

The reward points that the participants earned by repeating the course and the awards they received in return for these points are shown in Table 5. After the game, V5 earned 2 Colonel Badge points and V9 earned 3 Colonel Badge points.

The relationship between T-score, Roundtime and KeyStroke was expressed by calculating the Pearson correlation between the means of these values (tsmean, rtmean, ksmean) and presented in Table 6. It shows that there is a high negative correlation between T-score and Roundtime and Keystroke values. Each replay causes a decrease in the time spent per round in the game and a decrease in the need for keystrokes. In addition, there is a high positive correlation between Keystroke and Roundtime values.

**Figure 6:**
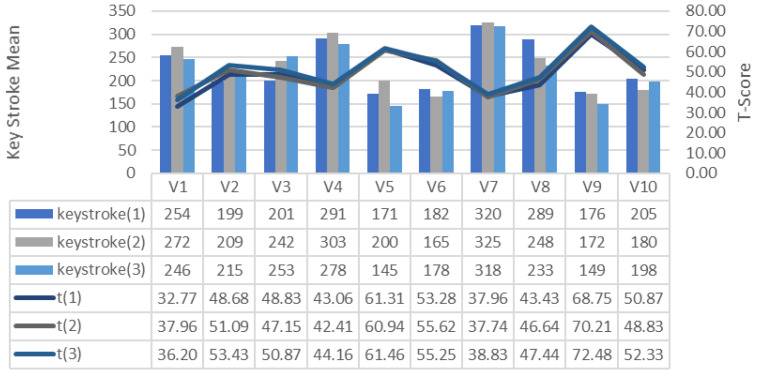
Relationship between the number of keystrokes per round on the track GDES and game performance (T-Score).

The number of keystrokes of the commands given by the participants for drone control in each training session is shown in Figure 6. It is observed that as the game performance of the participants increases, the keystrokes decrease. This finding suggests that high-performance players have shorter game completion times on average and require fewer keystrokes in parallel. The minimum number of keystrokes belongs to the V5, which is 145 in the 3rd training, and the average of the V5 is 172. The average number of keystrokes of the V9 with the highest game performance average is 166.

**Figure 7:**
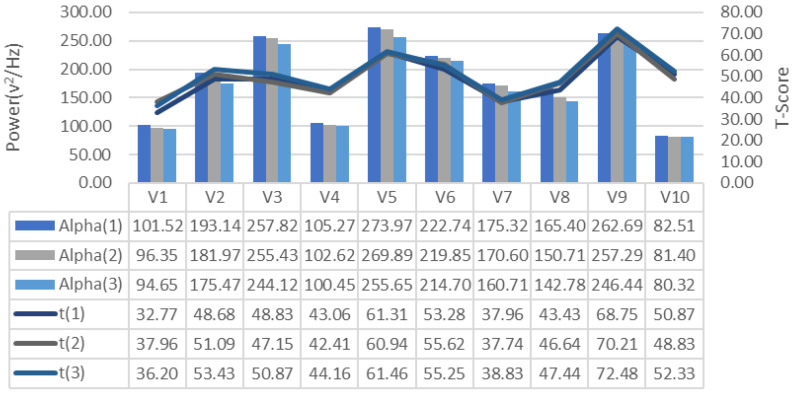
GDTS per round on track with Alpha band power game performance (T-Score) relationship.

**Figure 8:**
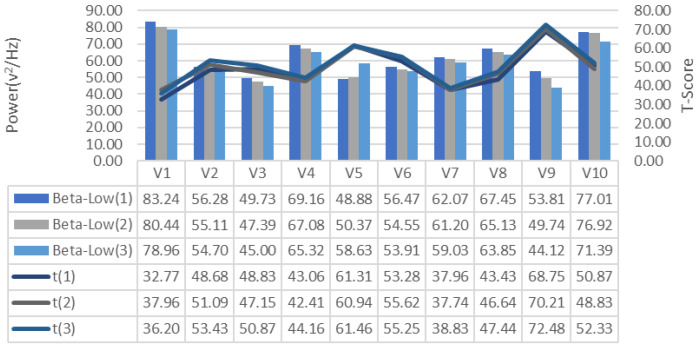
GDTS per round on track with Beta-Low band power game performance (T-Score) relationship.

**Figure 9:**
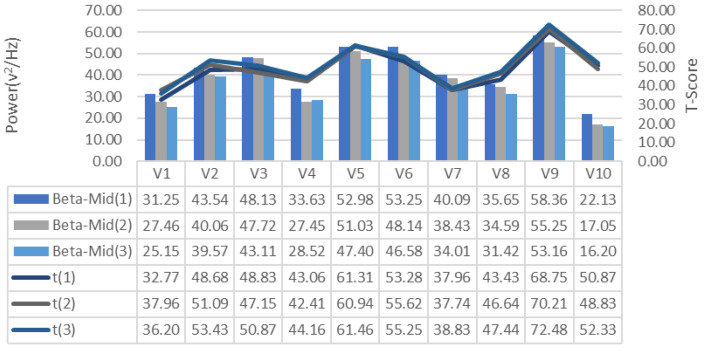
GDTS per round on track with Beta-Mid band power game performance (T-Score) relationship.

**Figure 10:**
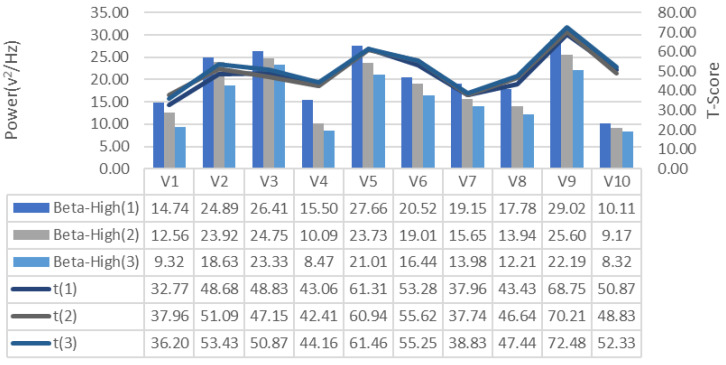
GDTS per round on track with Beta-High band power game performance (T-Score) relationship.

**Figure 11:**
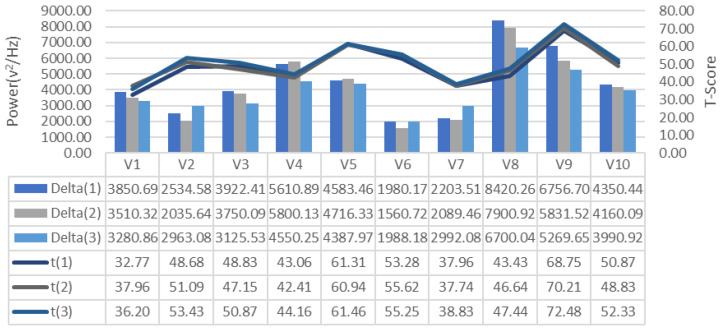
GDTS per round on track with Delta band power game performance (T-Score) relationship.

**Figure 12:**
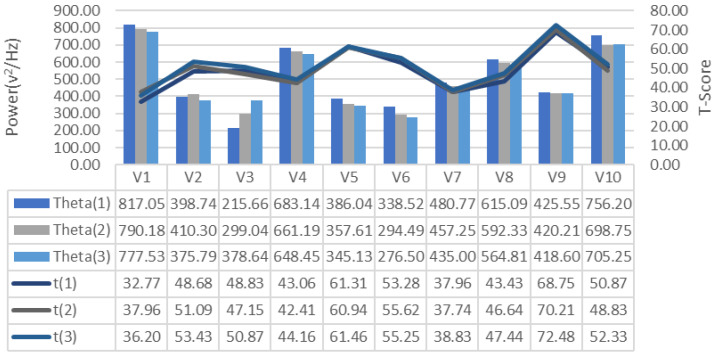
GDTS per round on track with Theta band power game performance (T-Score) relationship.

EEG signals collected during the training process were analyzed for frequency bands and changes in alpha, beta-low, beta-medium, beta-high, delta and theta bands recorded for each repeated GDTS track training of each subject, respectively Figure 7, Figure 8 and Figure 9, is shown in Figure 10, Figure 11 and Figure 12. When the changes related to the bands are examined, it is seen that there is a decrease in the strength of the alpha band and all beta bands in parallel with the increase in the number of repetitions of the tracks.

It is observed that the alpha band power of the participants who complete the task quickly is higher than the other participants, and the participants who complete the task slowly, on the contrary, have low alpha band power. It is seen that Beta-Low band power is higher in V1 and V10. This shows that these participants are more relaxed and focused than other participants. Increases in Alpha and Beta-Low bands indicate relaxation and formerly relaxed state, respectively [47]. Accordingly, it can be inferred that V1 is not comfortable compared to other participants, and V9 is more comfortable. In addition, it has been determined that the Beta-Mid and Beta-High band power of V9 and V5 with high GDTS track performances are higher than the others.

**Figure 13:**
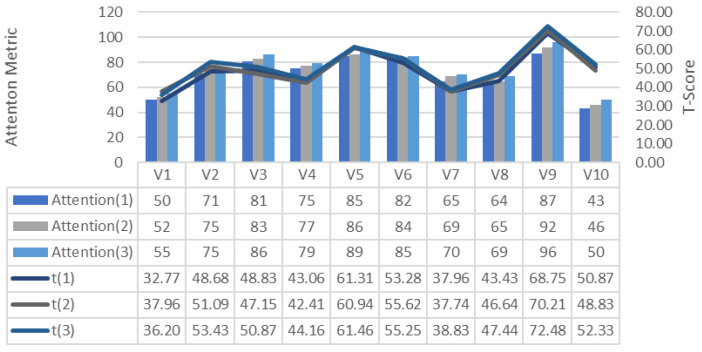
Change in attention per lap on the GDTS track.

**Figure 14:**
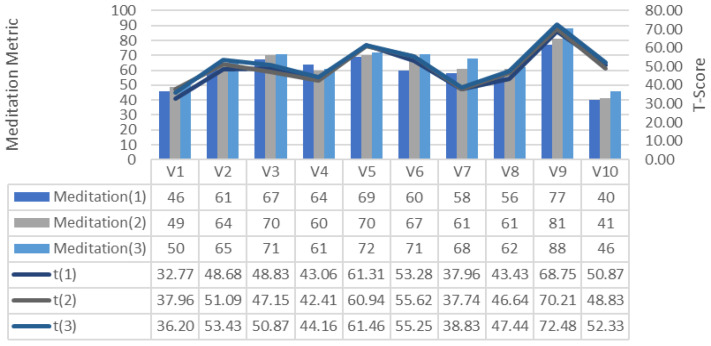
Change in the meditation per tour on the GDTS track.

Figure 13 and Figure 14 show the changes in the GDTS tracks of attention and meditation emotion measurements generated from the participants’ brain signals. These values show that attention and meditation increase with the increase in the number of trainings in GDTS tracks. This can be explained as the greater number of training sessions, the more focused the participants on the game and their attention.

**Figure 15:**
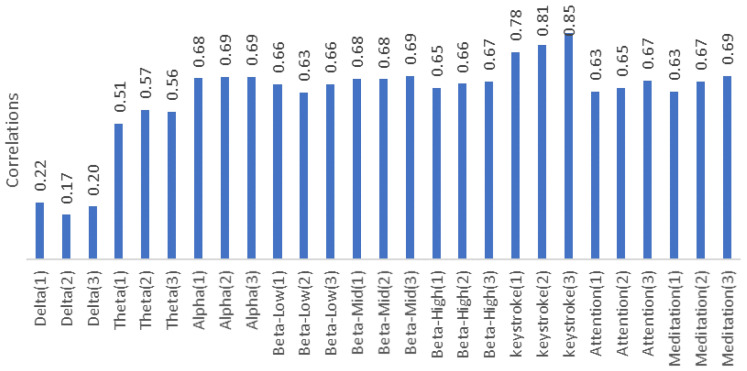
Game performance (T-score) correlation of EEG data and number of keystrokes per round in the GDTS track.

The absolute values of the Pearson cross correlation coefficient for Delta, Theta, Alpha, Beta-Low, Beta-Mid, Beta-High and Keystroke values are shown in Figure 15. The correlation values of Theta, Beta-Low and Keystroke values are negative, but the values in Figure 15 are absolute values. The highest correlation among the EEG bands is the Alpha and Beta-Mid bands with an average value of 0.69. The delta band appears to be unrelated to score. Keystroke values appear to be highly correlated with T-score. The delta band appears to be slightly correlated and the Theta band negatively related. In the study, it is seen that as the number of trials increases, the subjects complete their lessons in a shorter time and the number of keystrokes decreases slightly with the increase in the number of trials. This can be seen because of training sessions at GDTS. It can be thought that as the participants become more familiar with the commands, they start pressing fewer keys. In the research, as the number of repetitions of the track increases; it is observed that the number of keystrokes per task of the participants decreased in 70% of the participants. The reduction in the number of keystrokes can be seen because of the participants making fewer keystrokes as they become accustomed to commands, and the tracks in GDTS being unchanged.

## 4. Conclusions

In this study, an objective measurement system that can be used in computer aided education systems is proposed. For this purpose, a multimodal recording system that can simultaneously collect keyboard and brain activity data during simulator use has been developed to analyze the cognitive and physical development of students during drone pilot training. Using this system, it has been determined that the number of keystrokes is generally lower in participants with high gaming performance than other participants. In addition, it is seen that attention and meditation values are higher with alpha, beta mid and beta high band strength. This result shows that game performance is related to the stated characteristics and revealed that the height of attention and meditation ensures that game performance and learning are more effective [48].

As the number of trials increased, it was observed that the alpha and beta band power decreased, and the meditation and attention values increased as the participants got used to the cognitive load in visual/auditory tasks. The repetition of the game reduced the stress and anxiety levels of the participants and enabled them to play more carefully. As a result, gaming performance has been positively affected. In addition, as the number of track repetitions increased, the task completion time of the participants decreased. With the increase in the number of track repetitions, it is seen that the commands given by the participants for task completion generally decrease. This showed that participants were more familiar with commands using GDTS, and increased game repetition was associated with increased reaction speed, reduced stress levels, and improved hand-eye coordination [35].

It is seen that this proposed system is an innovative system in the measurement of physical and mental activity. In order to find the ideal number of training in future studies, the number of participants, tasks and training should be increased. In addition, in future studies, each track will be differentiated from each other in terms of design (such as differentiating the objects on the track in terms of location, color and mobility) and difficulty, and the tracks will be randomly assigned. Thus, it will be possible to evaluate the different game experiences of the players with these differences.

## Figures and Tables

**Table 1:** Studies using bio signals in gaming and education environments.

Ref.	Subject of Research	The Number of Participants	Research Measurement Method	Data Analysis Method
[32]	Determination of correlation between students’ attention levels and participation to lesson	21	Neurosky MindWave EEG	EEG signals were processed with the Fourier Transform method. The processed data were analyzed through Python and Matlab programs. In additon, the Pearson correlation coefficient was used to find the relationship between students’ attention levels and their participation in the lesson.
[34]	Determining the effect of game-based learning on the learning process	42	MRI and fMRI	T-tests were used in behavioral analysis, and multiple voxel model analysis (MVPA) was used in imaging analysis.
[35]	Determining the effects of action video games on players’ processing speed, hand-eye coordination, reaction time	5	EEG	Empirical mode decomposition was used for feature extraction from EEG data. K-Nearest Neighbor and Linear Discriminant Analysis were used to classify the data.
[36]	Estimation of emotion intensity in different games	12	Heart rate signals (HR), Facial Expressions (FE)	Bidirectional long and short term memory (Bi-LSTM) Network was used for teaching heart rate (HR) properties, Convolutional Neural Networks (CNN) for teaching facial expressions (FE), SOM-BP Network was used to combine HR and FE features.
[37]	Detection of emotions from multiple biosignals in virtual reality game	30	Electroencephalography (EEG), Galvanic skin response (GSR), Heart rate (HR)	Raw signals were standardized by the score normalization technique. Fast Fourier Transform technique was used to perform spectral analysis on the signals.

**Table 2:** Command and keys to control of drone.

Command	Key
Forward	W
Backward	S
Left	A
Right	D
Up	Up Arrow
Down	Down Arrow
Rotate Left	Q
Rotate Right	E
Down Camera Photo	Y
Forward Camera Photo	H

**Table 3:** EEG frequency bands and conditions.

Type	Frequency (Hz)	Mental State and Conditions
Delta	0.1–3	Deep, dreamless sleep, non-REM sleep or unconscious
Theta	4–7	Intuitive, creative, recall, fantasy, imaginary, dream
Alpha	8–12	Relaxed (but not drowsy) tranquil, conscious
Beta-Low	12–15	Formerly SMR, relaxed yet focused, integrated
Beta-Middle	16–20	Thinking, aware of self and surroundings
Beta-High	21–30	Alertness, agitation
Gamma	>31	Motor functions

**Table 4:** Participant Information.

Id	Age	Education	Game Experience
V1	25	Bachelor Degree	No
V2	32	Doctoral Degree	No
V3	18	Bachelor Degree	No
V4	21	Bachelor Degree	Yes
V5	34	Doctoral Degree	No
V6	23	Bachelor Degree	Yes
V7	21	Bachelor Degree	Yes
V8	34	Doctoral Degree	Yes
V9	28	Bachelor Degree	No
V10	30	Master Degree	Yes

**Table 5:** Participants game points and rewards chart.

	V1	V2	V3	V4	V5	V6	V7	V8	V9	V10
Repetition-I	100	400	400	300	800	500	200	300	900	500
Repetition-II	200	500	400	300	700	600	200	400	1000	400
Repetition-III	100	500	500	300	800	600	200	400	1000	500
Track Award	-	-	-	-	2 Colonel badges	-	-	-	3 Colonel badges	-
Provisions for Awards	-	-	-	-	20 TL	-	-	-	1 General badge = 50 TL	-

**Table 6:** Representation of the relationship between T-score, Round Time, Keystroke with Pearson’s correlation.

	Ksmean	Rtmean	Tsmean
ksmean	1.00		
rtmean	1.00	1.00	
tsmean	−0.99	−1.00	1.00

## Data Availability

The datasets generated during and/or analyzed during the current study are available from the corresponding author on reasonable request.
